# Na_V_1.4 DI-S4 periodic paralysis mutation R222W enhances inactivation and promotes leak current to attenuate action potentials and depolarize muscle fibers

**DOI:** 10.1038/s41598-018-28594-5

**Published:** 2018-07-10

**Authors:** Landon Bayless-Edwards, Vern Winston, Frank Lehmann-Horn, Paula Arinze, James R. Groome, Karin Jurkat-Rott

**Affiliations:** 1 Department of Biological Sciences, Idaho State University, 83209 Pocatello, ID USA; 2 Department of Applied Physiology, Ulm University, 89081 Ulm, Germany; 3 Department of Neuroanesthesiology, Clinic for Neurosurgery, Ulm University, Guenzburg, Germany

## Abstract

Hypokalemic periodic paralysis is a skeletal muscle disease characterized by episodic weakness associated with low serum potassium. We compared clinical and biophysical effects of R222W, the first hNa_V_1.4 domain I mutation linked to this disease. R222W patients exhibited a higher density of fibers with depolarized resting membrane potentials and produced action potentials that were attenuated compared to controls. Functional characterization of the R222W mutation in heterologous expression included the inactivation deficient IFM/QQQ background to isolate activation. R222W decreased sodium current and slowed activation without affecting probability. Consistent with the phenotype of muscle weakness, R222W shifted fast inactivation to hyperpolarized potentials, promoted more rapid entry, and slowed recovery. R222W increased the extent of slow inactivation and slowed its recovery. A two-compartment skeletal muscle fiber model revealed that defects in fast inactivation sufficiently explain action potential attenuation in patients. Molecular dynamics simulations showed that R222W disrupted electrostatic interactions within the gating pore, supporting the observation that R222W promotes omega current at hyperpolarized potentials. Sodium channel inactivation defects produced by R222W are the primary driver of skeletal muscle fiber action potential attenuation, while hyperpolarization-induced omega current produced by that mutation promotes muscle fiber depolarization.

## Introduction

Familial hypokalemic periodic paralysis (HypoPP) is an autosomal dominant skeletal muscle disease characterized by episodes of flaccid muscle weakness in response to decreased serum potassium. Spontaneous episodes typically begin in the first two decades of life, triggered by hypokalemia induced by insulin secretion following carbohydrate-rich meals, glucocorticoids from stress or infection, or potassium reuptake in muscle at rest after strenuous work. Nearly all mutations causing familial HypoPP are found in voltage-gated ion channels of skeletal muscle, such as the hCa_V_1.1 calcium channel (encoded by *CACNA1S*) in type I HypoPP, or the homologous hNa_V_1.4 sodium channel (encoded by *SCN4A*) in type II HypoPP^1,2^. Mutations in inwardly-rectifying potassium channels have been identified in non-familial forms of periodic paralysis^3^. Voltage-gated ion channels share a common functional architecture, with a voltage sensor module (S1-S4 segments) and a pore module (S5-S6 segments). Neutralization of positively charged arginine residues in voltage-sensing S4 segments is a recurrent pattern for HypoPP mutations in calcium and sodium channels. These S4 alpha helices move outwardly with membrane depolarization and induce voltage-sensitive opening of the central conducting alpha pore.

In type II HypoPP, generalized sodium channel gating defects include reduced current density and enhanced fast or slow inactivation (reviewed in^4^). These findings do not explain why muscle fibers from patients exhibit a depolarization of membrane potential in low extracellular potassium^5–7^. Several studies have implicated an aberrant “omega current” through the voltage sensor module as a contributing factor towards this paradoxical depolarization (reviewed in^1,4,8,9^). Recent models predict that at resting potential, the outermost S4 arginine (R1) separates the extracellular space from cytoplasm by occluding a narrow constriction in the voltage sensor. This constriction, termed the gating charge transfer center or ‘gating pore’, is lined with a conserved aromatic S2 residue and conserved negative countercharges in S2 and S3. As the gating pore is sequentially occupied by arginines in the second and third positions in S4 (R2 and R3) with depolarization, transient interactions are made between these positively charged S4 residues and the residues of the gating charge transfer center^8,10^. Mutations that replace the bulky guanidyl side chain of an S4 arginine with a residue containing a smaller side chain abolish the separation between the cytoplasm and extracellular fluid and cause a proton- or cation-selective omega current that flows through the gating pore constriction. Mutations at R1 or R2 produce an omega current with membrane hyperpolarization^11–13^, while mutations at R3 produce this current with depolarization^14,15^. Thus, HypoPP S4 mutations may affect not only the voltage sensitivity and amplitude of the central alpha pore current, but also uncover an omega gating pore current that contributes to the clinical features of the channelopathy.

To date, S4 mutations associated with type II HypoPP have been identified in domains DI - DIII of hNa_V_1.4^16^. The first DI-S4 mutation associated with HypoPP (R222W) was reported at R2^16,17^. Affected family members presented with a clinical phenotype of respiratory insufficiency during severe weakness, with a fatal outcome of one member. Here, we report an additional family with the R222W mutation and characterize its effects in patient muscle fibers, in heterologous expression, and with computational simulations. Results from this combined approach to the investigation of R222W show that the mutation attenuates action potentials in patient muscle fibers by enhancing sodium channel inactivation and depolarizes muscle fibers by promoting an inwardly directed omega current. Our findings extend the investigation of HypoPP sodium channel mutations on skeletal muscle fiber excitability to include DI-S4 and are discussed with respect to the function of this channel region.

## Results

### Patients

The 52-year-old male proband had spontaneous morning paralytic attacks of 2-3 hours in duration, typically in the upper extremities. Complete tetraplegia occurred at age 18 and again at age 19. The weakness spells were associated with mild hypokalemia, 3-fold increased creatine kinase values, and myalgias that outlasted the weakness by several hours. Onset was at age 18, and frequency of attacks was once quarterly. The patient reported cold environment as a triggering factor. Additionally, a carbohydrate-rich meal on the ward at night triggered a weakness spell in the morning. Thyroid hormone levels were normal. An EMG of the tibialis anterior muscle showed a mild myopathic pattern but no pathologic spontaneous activity. The histology of the latissimus dorsi muscle revealed mild type 2 single fiber atrophy. Electron microscopy showed a proliferation of the sarcoplasmic reticulum but no tubular aggregates. Because of the predominant myalgias, muscle activities of forward and backward carnitine palmityl transferase were measured and were found normal, as were all routinely determined muscular enzyme activities. Molecular genetics identified a heterozygous hNa_V_1.4-R222W mutation (*SCN4A*-C664T) in the patient and three family members without history of weakness episodes (two females, one male).

### *In-vitro* studies on native muscle fibers

**Figure 1:**
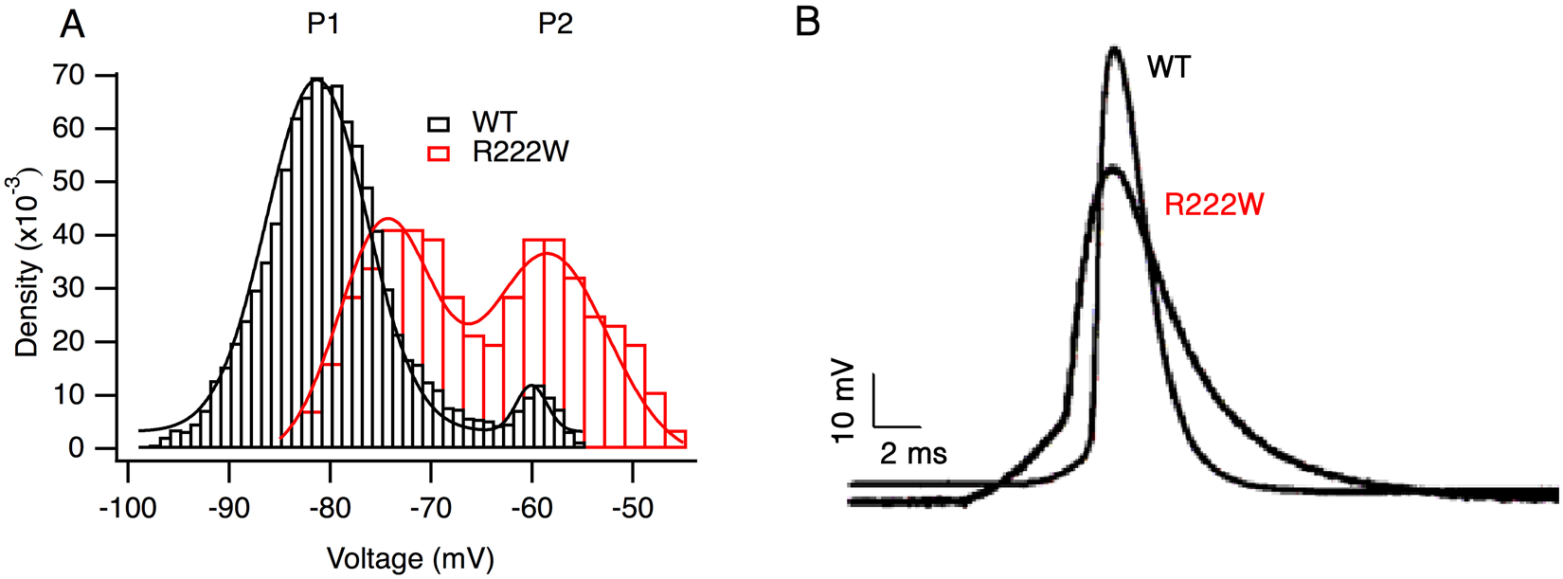
R222W affects resting and action potentials in muscle fibers. The density of muscle fibers at various resting membrane potentials shows peaks at P_1_ (hyperpolarized) and P_2_ (depolarized) for control and R222W (**A**). P_1_ peaks are −80.9 ± 0.1 mV (control) and −74.5 ± 0.4 mV (R222W), with P_2_ peaks at −60.2 ± 0.4 mV (control) and −58.4 ± 0.4 mV (R222W). Action potentials in R222W (n = 10) fibers have a maximum amplitude of −8 ± 5 mV, significantly (P ≤ 0.05) less than that for control fibers (n = 6) at +11 ± 8 mV (**B**).

In bath solution containing 4 mM extracellular K^+^, excised control or patient R222W muscle fibers showed a bimodal distribution of resting membrane potentials (Fig. 1A). Gaussian fitting revealed peak values of P_1_ = −74.5 ± 0.4 mV in R222W (χ^2^ = 1.2 × 10^−5^), which were depolarized compared to control fibers (−80.9 ± 0.1 mV; χ^2^ = 4.0 × 10^−6^) and P_2_ values which were similar (−58.4 ± 0.4 mV for R222W and −60.2 ± 0.4 mV for controls). A larger fraction of patient fibers populated the P_2_ state compared to control fibers, suggesting an increased tendency to depolarize. Here, the respective densities for P_1_ and P_2_ were 0.56 and 0.52 in patient fibers, compared to 0.80 and 0.04 for control fibers. Decreasing the extracellular K^+^ to 1 mM severely depolarized patient muscle fiber potentials to a single peak at −50.0 mV ± 0.4 mV (data not shown). In contrast, control fibers in 1 mM K^+^ reveal a trimodal distribution^15^, with peaks at −89.7 ± 0.6, −72.7 ± 0.2, and −52.2 ± 0.5 mV, and with a larger fraction of muscle fibers at the more negative membrane potentials (respective densities of 0.14, 0.68, 0.25).

Action potentials of fibers harboring the R222W mutation showed undershoots of −8 mV ± 5 mV (n = 10) instead of overshoots as in control fibers (+11 ± 8 mV, n = 6, P ≤ 0.05) in accordance with a partially inactivated sodium channel population (Fig. 1B). The maximum rates of rise of R222W action potentials elicited from a holding potential of −80 mV were 0.2 ± 0.03 V/ms (n = 10) and therefore about 30% less than those from control fibers (0.3 ± 0.04 V/ms, n = 6, P ≤ 0.01, data not shown), also suggesting a reduced sodium channel availability.

### Cut-open oocyte recordings: gating parameters

**Figure 2:**
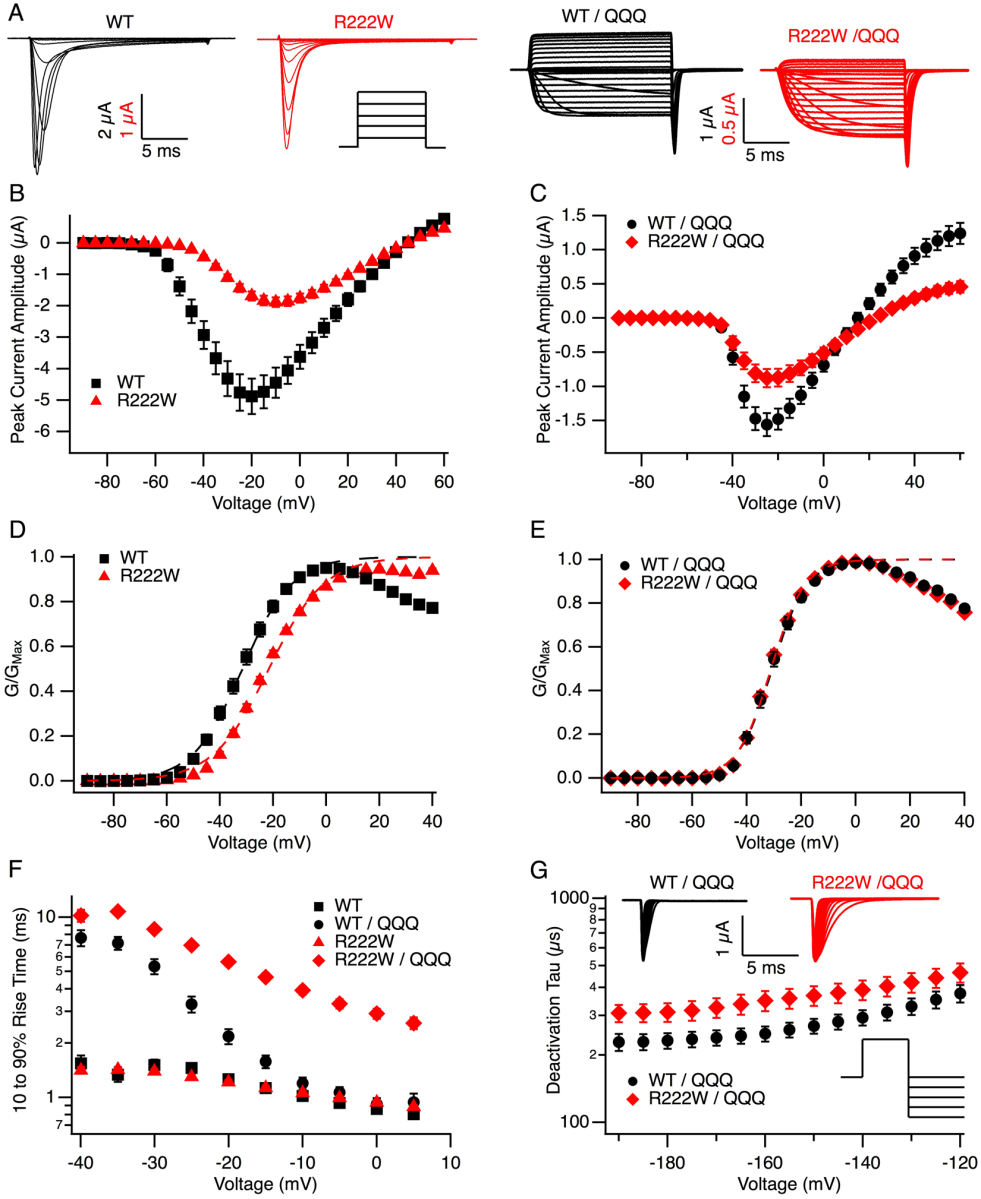
R222W slows activation and deactivation. Traces of sodium currents in response to step depolarizations from −90 to +60 mV for 20 ms are shown in (**A**), along with a schematic of the protocol for activation. I/V relationships are plotted for WT (n = 126) and R222W (n = 73) in (**B**) and compared in (**C**) utilizing the IFM/QQQ background (n = 17 and n = 21, respectively). G/V relationships fit to a Boltzmann function with the asymptote held at one are shown in (**D**, native background) and (**E**, IFM/QQQ background). R222W produces a +10.4 mV shift compared to WT in the native (n = 81 and n = 88, respectively), but not IFM/QQQ background (n = 63 and n = 48, respectively). Times for 10–90% rise to peak activation are shown in (**F**) for WT and mutant channels with the native (n = 48 and n = 73, respectively) or IFM/QQQ background (n = 43 and n = 45, respectively). Activation is slowed by R222W in the absence of IFM-mediated inactivation. Deactivation time constants are shown in (**G**) for WT/QQQ (n = 26) and R222W/QQQ (n = 29). R222W/QQQ slows deactivation as determined from tail currents (shown in inset) elicited by depolarizing channels to the empirical reversal potential for 30 ms, followed by 20 ms hyperpolarizing commands from −190 to −120 mV. Values are shown in Table 1.

To determine whether hypoexcitability associated with the R222W mutation results from defects in sodium channel inactivation or depolarizing omega current, we characterized biophysical properties of mutant and wild type hNa_V_1.4 channels in heterologous expression, with results summarized in Table 1. R222W decreased peak current amplitude compared to wild type (P < 0.01; Fig. 2B) and significantly decreased the probability of activation, as indicated by a 10 mV depolarizing shift of G/V midpoint, and a significantly reduced slope (both P < 0.0001; Fig. 2D). In contrast, R222W did not affect activation kinetics calculated from 10–90% rise time (P = 0.3 at −10 mV; Fig. 2F).

**Table 1:** Summary of electrophysiology results. Gating parameters obtained from WT or R222W channels expressed in *Xenopus* oocytes at 20 °C.

Parameter	WT		R222W		Statistical Significance
	Mean ± SEM	*N*	Mean ± SEM	*n*	
**Peak Current Amplitude**					
Native (µA)	−4.9 ± 0.6	126	−1.9 ± 0.2	73	U_197_ = 11515, P < 0.01
IFM/QQQ (µA)	−1.6 ± 0.1	17	0.9 ± 0.1	21	t_36_ = 3.23, P = 0.003
**Activation Native**					
Conductance midpoint (mV)	−32.3 ± 1.2	88	−22.6 ± 0.7	81	U_167_ = 1676, P < 0.0001
Conductance slope	4.8 ± 0.1	88	3.0 ± 0.1	81	U_167_ = 774, P < 0.0001
10–90% Rise Time τ −10 mV (ms)	1.0 ± 0.04	48	1.1 ± 0.03	73	t_119_ = 0.92, P = 0.3
**Activation IFM/QQQ**					
Conductance midpoint (mV)	−30.9 ± 0.9	48	−31 ± 0.6	63	U_109_ = 1453, P = 0.7
Conductance slope	5.8 ± 0.3	48	5.1 ± 0.2	63	U_109_ = 1208, P = 0.07
10–90% rise time τ −10 mV (ms)	1.2 ± 0.1	43	3.9 ± 0.2	45	U_86_ = 29, P < 0.0001
**Deactivation IFM/QQQ**					
τ at −160 mV (ms)	0.3 ± 0.03	26	0.4 ± 0.04	29	U_53_ = 239, P < 0.05
**Steady-State Fast Inactivation**					
Midpoint (mV)	−59.4 ± 0.8	61	−86.7 ± 0.9	53	t_112_ = 23.7, P < 0.0001
Slope	5.1 ± 0.1	61	4.3 ± 0.1	53	U_112_ = 718, P < 0.0001
Open-state τ −20 mV (ms)	1.7 ± 0.2	53	1.1 ± 0.03	50	U_101_ = 920.5, P < 0.01
Closed-state τ −65 mV (ms)	22.7 ± 1.3	28	25.3 ± 2.3	40	U_66_ = 526, P = 0.7
Recovery τ −100 mV (ms)	2.27 ± 0.02	17	41.4 ± 5.9	5	U_20_ = 0.000, P < 0.0001
**Slow Inactivation**					
Steady-state midpoint (mV)	−54.9 ± 2.9	32	−96.1 ± 2.1	34	U_64_ = 1043, P < 0.0001
Steady-state slope	0.8 ± 0.08	32	1.5 ± 0.1	34	U_64_ = 965, P < 0.0001
Onset τ (s)	28.2 ± 4.8	22	36.9 ± 8.6	17	U_37_ = 175, P = 0.7
Recovery τ (s)	6.5 ± 0.9	25	37.1 ± 6.6	27	U_*50*_ = 67, P < 0.0001

The inactivation deficient IFM/QQQ background^18^ was used to isolate activation and deactivation from indirect effects of fast inactivation on measured sodium currents. R222W/QQQ significantly decreased peak current amplitude (P < 0.01; Fig. 2C) but was without effect on activation probability compared to hNa_V_1.4/QQQ (P = 0.7 for midpoint, P = 0.07 for slope; Fig. 2E). However, activation and deactivation kinetics were each slowed by R222W compared to hNa_V_1.4 in the IFM/QQQ background (activation at −10 mV, P < 0.0001; Fig. 2F; deactivation at −160 mV, P < 0.05; Fig. 2G).

**Figure 3:**
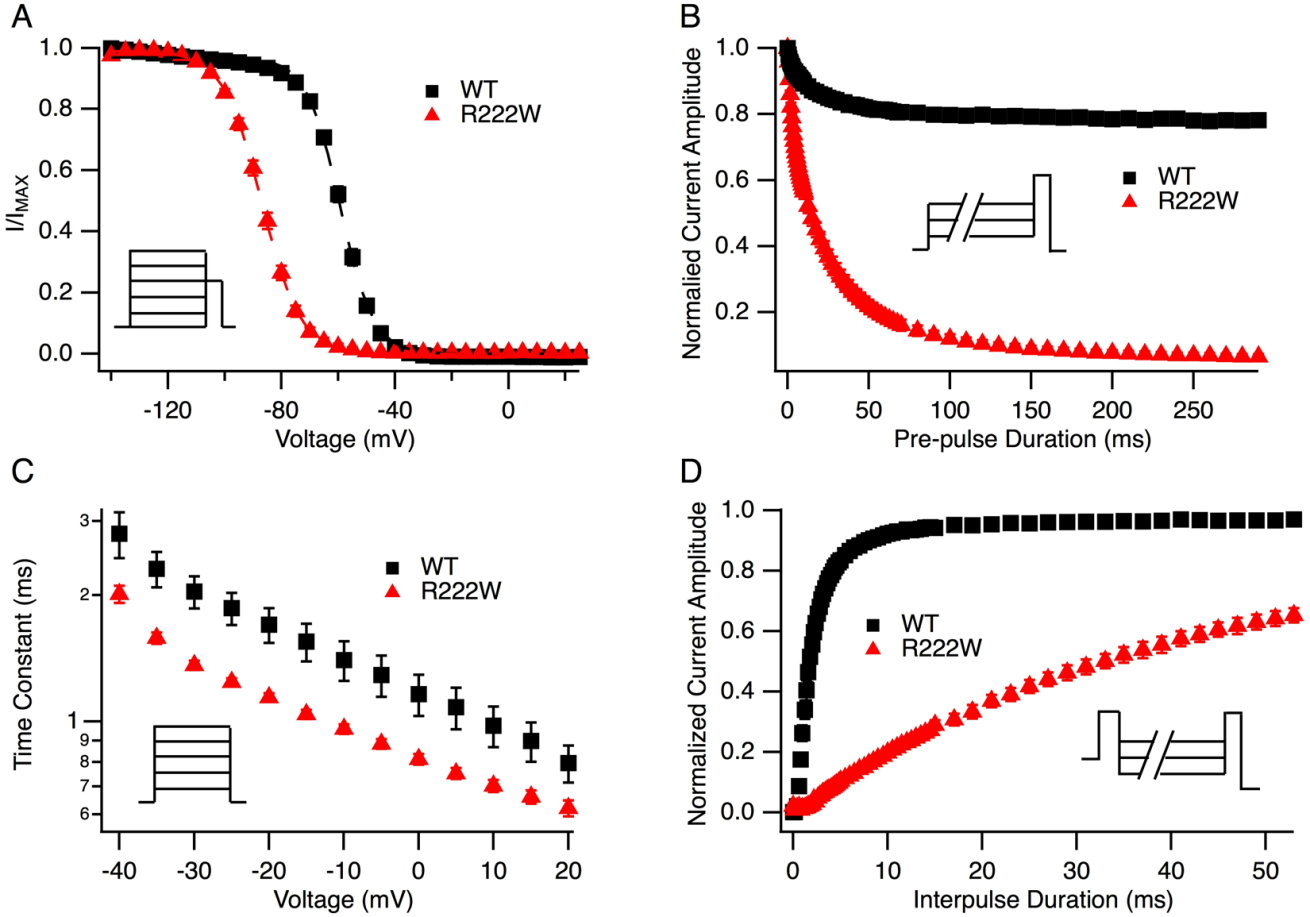
R222W stabilizes the fast-inactivated state. Steady-state fast inactivation is shown in (**A**). Channels were conditioned for 300 ms from −140 mV to +20 mV, and channel availability tested with 20 ms, −20 mV test pulses (inset). R222W (n = 53) produces a −27 mV shift in the midpoint of the steady state fast inactivation curve compared to WT (n = 61). Time course of closed-state inactivation is shown in (**B**) for a variable-duration conditioning potential of −65 mV (protocol used shown in inset); R222W (n = 40) elicits more complete inactivation than WT (n = 28). Onset of fast inactivation from the open state is shown in (**C**). Compared to WT (n = 53), R222W (n = 50) accelerates open-state fast inactivation as determined from the decay of sodium currents in response to 20 ms step depolarizations from −40 mV to +20 mV. Recovery from fast inactivation is shown in (**D**). R222W (n = 5) slows recovery compared to WT (n = 17), measured by a double pulse protocol in which channels were inactivated for 100 ms and hyperpolarized (−100 mV shown) for increasing durations, followed by a 20 ms, −20 mV test pulse to test channel availability. Values are shown in Table 1.

The observed decrease in probability of activation in R222W channels might be due to enhanced fast inactivation. R222W produced a 27 mV hyperpolarizing shift in the midpoint of the steady-state fast inactivation curve, and with a decreased slope (both P < 0.0001; Fig. 3A). Closed-state fast inactivation in R222W and wild type hNa_V_1.4 are compared at −65 mV in Fig. 3B. While R222W elicited greater completion than hNa_V_1.4 at this voltage (91 ± 0.8% *versus* 21 ± 1.6%, P < 0.0001), kinetics of closed-state entry into fast inactivation were similar (P = 0.7). R222W accelerated open-state entry into fast inactivation (Fig. 3C) and slowed recovery (−100 mV, P < 0.0001; Fig. 3D).

**Figure 4:**
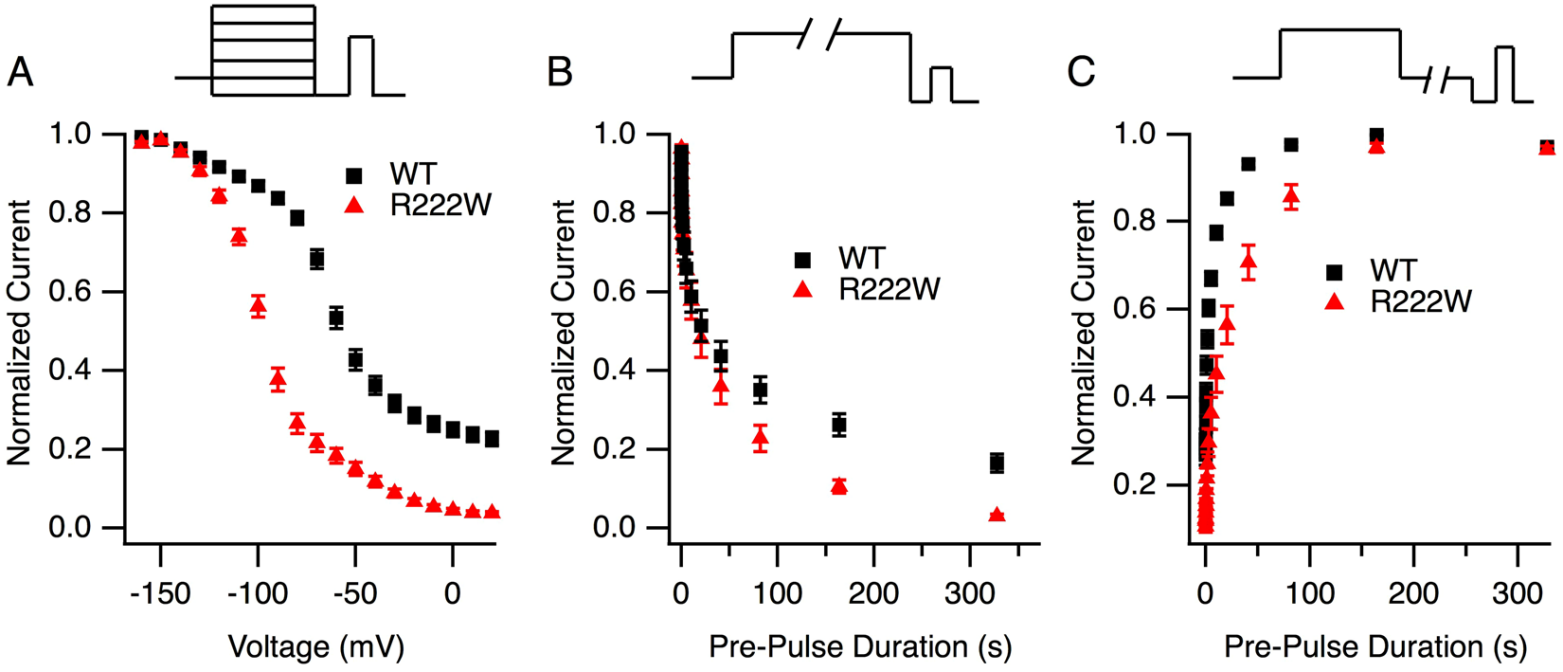
R222W enhances slow inactivation. In all experiments, channels were recovered from fast inactivation at −120 mV (20 ms) prior to a test pulse at −20 mV to assess channel availability. Steady-state slow inactivation is shown in (**A**) from experiments in which channels were conditioned to voltages shown for 90 s prior to test. R222W (n = 34) significantly increases the completion and probability of slow inactivation compared to WT (n = 32). In (**B**), onset of slow inactivation was determined from loss of channel availability following a variable duration (0 to 360 s) depolarization to 0 mV. R222W (n = 17) did not significantly affect onset of slow inactivation compared to WT (n = 22). For recovery (**C**), a 0 mV, 90 s command to promote slow inactivation was followed by variable duration (0 to 360 s) hyperpolarization at −120 mV and test. R222W (n = 27) slows recovery from slow inactivation compared to WT (n = 25). Values are shown in Table 1.

Slow inactivation parameters were determined as putative, additional gating defects for the R222W mutation. Slow inactivation in hNa_V_1.4 reached 77 ± 2% completion (Fig. 4A), consistent with patch-clamp studies of this isoform expressed in oocytes^19^. R222W increased completion of slow inactivation to 96 ± 0.5% (P < 0.0001), produced a 41 mV hyperpolarizing shift of midpoint, and with increased slope (both P < 0.0001). Whereas onset of slow inactivation was similar in R222W and hNa_V_1.4 (Fig. 4B), R222W prolonged the recovery from slow inactivation (37 ± 7 s *versus* 6.5 ± 1 s, P < 0.0001; Fig. 4C). Taken together with the above results, our findings indicate that the R222W mutation stabilizes sodium channel inactivation and suggest that these effects are casual to the hypoexcitable phenotype.

### Cut-Open Oocyte Recordings: omega current

**Figure 5:**
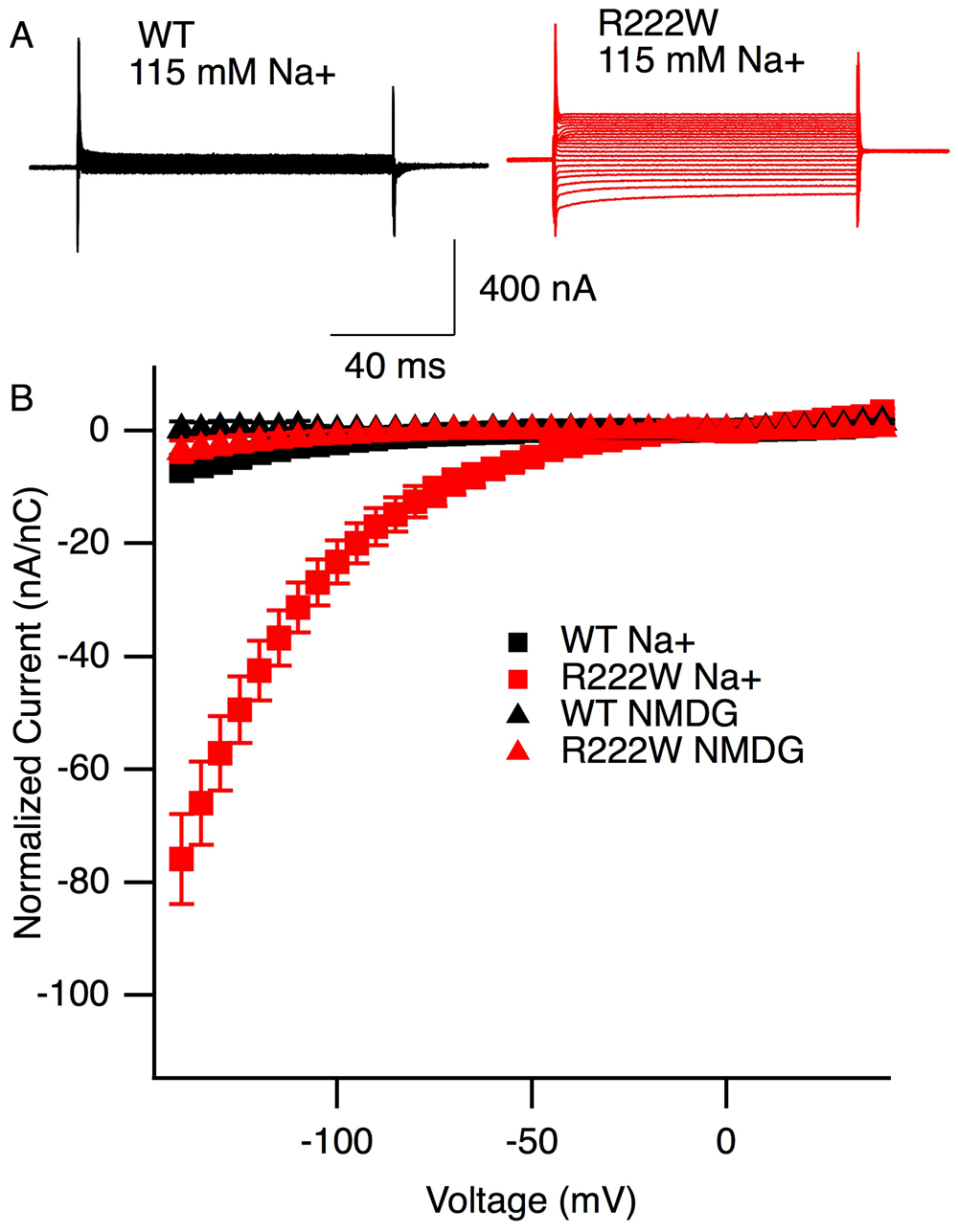
R222W promotes a cationic leak current. Current traces from TTX-blocked channels in response to step commands to voltages ranging from −140 mV to +40 mV are shown in (**A**) for WT and R222W, with 115 mM Na^+^ in the external bath solution. Ohmic current from −30 mV to +20 mV was subtracted from raw current values and normalized against anodic gating current recorded in response to +40 mV step depolarization. Plot of the normalized leak current as a function of gating charge at +40 mV is shown in (**B**, n = 8 to 12). With 115 mM Na^+^ external, R222W channels show an inwardly directed current at hyperpolarized voltages, not observed for WT channels. Ohmic responses are observed for R222W or WT when NMDG is used as the external cation.

R222W, but not wild type hNa_V_1.4, produced an inwardly directed sodium current detected at voltages more negative than −40 mV (P < 0.01; Fig. 5). Omega current was not observed when Na^+^ was replaced by NMDG (N-methyl D-glucamine) as the relative cation in the external solution. Thus, the HypoPP mutation at DI-S4:R2 produced a cationic omega current in response to membrane hyperpolarization, as previously observed for type II HypoPP mutations at DII-S4:R2^13^ or at DIII-S4:R2^20^.

### Action potential modeling

**Figure 6:**
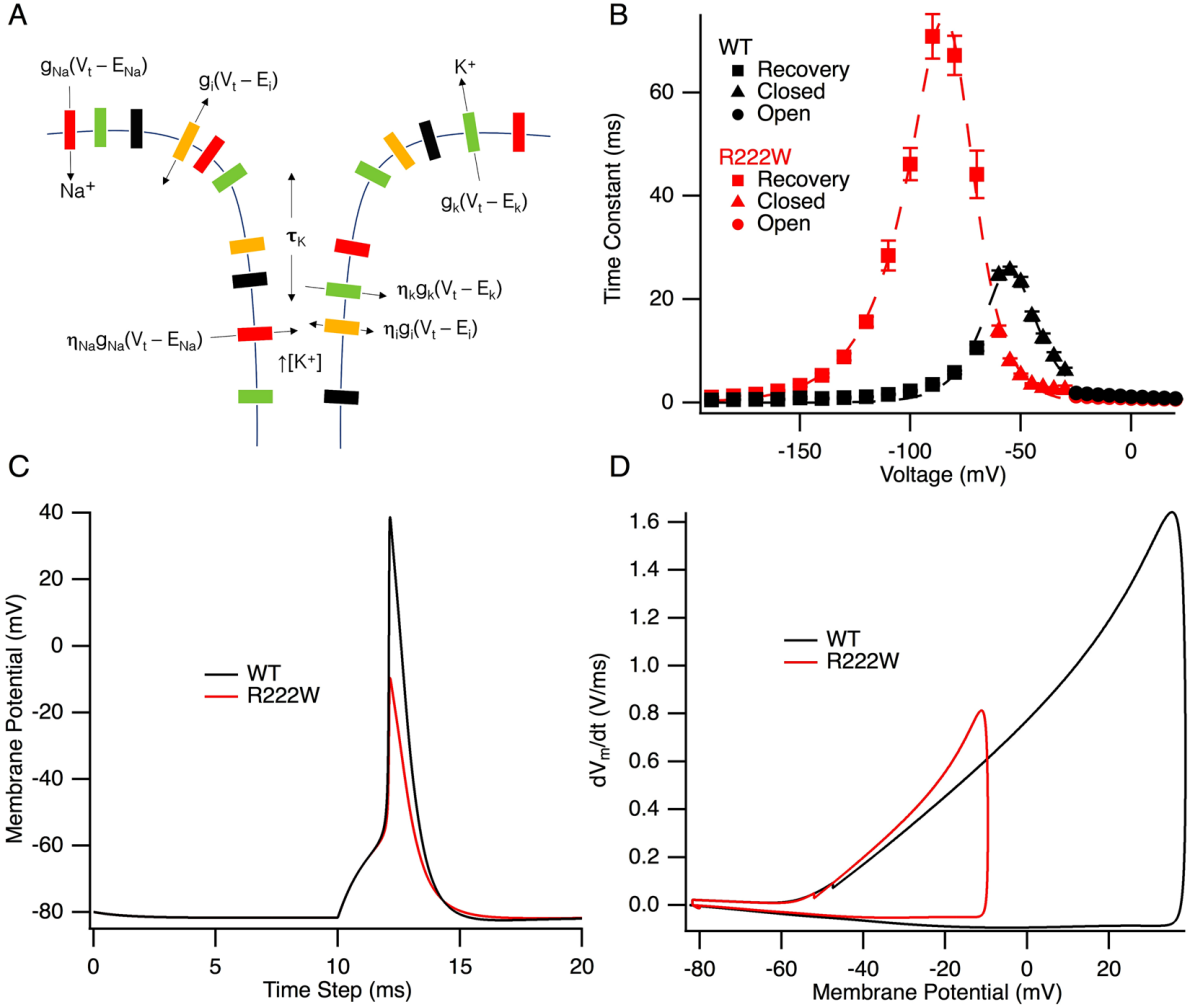
R222W action potential height is attenuated in simulation. A schematic of the main parameters used in the action potential model is shown in (**A**). The sarcolemma and t-tubule were both modeled, as well as potassium accumulation in the t-tubule. Barrier models of inactivation kinetics are shown in (**B**) for WT and R222W. Time constants were obtained at voltages from −190 mV to −70 mV (recovery), −60 mV to −30 mV (closed-state fast inactivation) and −25 mV to 20 mV (open-state fast inactivation). The fractional barrier distance is 0.50 for WT and 0.35 for R222W. The midpoint of the barrier is −55.1 mV for WT and −80.6 mV for R222W. These kinetic parameters were used to simulate wild type and R222W (attenuated) action potentials shown in (**C**), with peak height of the R222W action potential at −9.6 mV and that for WT at +38.7 mV. A phase diagram of the rate of voltage change is shown in (**D**). The peak rate of rise (0.8 V/ms) is 50% slower for R222W action potentials compared to WT (1.6 V/ms).

Summary time constants for the full range of voltages for entry into or recovery from the fast-inactivated state for hNa_V_1.4 or R222W are shown in Fig. 6B. Rate equations (Supplementary Table S2) were obtained from Gaussian fits to these curves and applied to the model as α_h_ and β_h_ parameters defining transition into, and exit from, the inactivated state. These empirically derived α_h_ and β_h_ constants were the only difference in parameters utilized to simulate WT and R222W action potentials. The simulated action potentials in R222W fibers showed an undershoot (peak height of −10 mV) compared to an overshoot in control fibers (peak height of +39 mV; Fig. 6C), a finding consistent with the observed effect of the mutation on action potentials recorded from patient muscle fibers. The phase diagram shows a slower peak rate of rise for R222W (0.8 V/ms) compared to WT (1.6 V/ms; Fig. 6D), also consistent with results from patient muscle fiber recordings.

### Molecular dynamics

We utilized molecular dynamics simulations to examine the effect of tryptophan substitution at R222 on transient gating charge transfer center interactions during hyperpolarization, as a possible mechanism for R222W-elicited omega current. Here, the focus of investigation was the interaction of the residue at DI-S4:R2/W2 with the aromatic tyrosine in DI-S2 (Y168), and negative countercharges in DI-S2 (E171) and DI-S3 (D197). These conserved residues in the gating charge transfer center have been shown to interact with S4 residues and may occlude the gating pore^10,21,22^.

**Figure 7:**
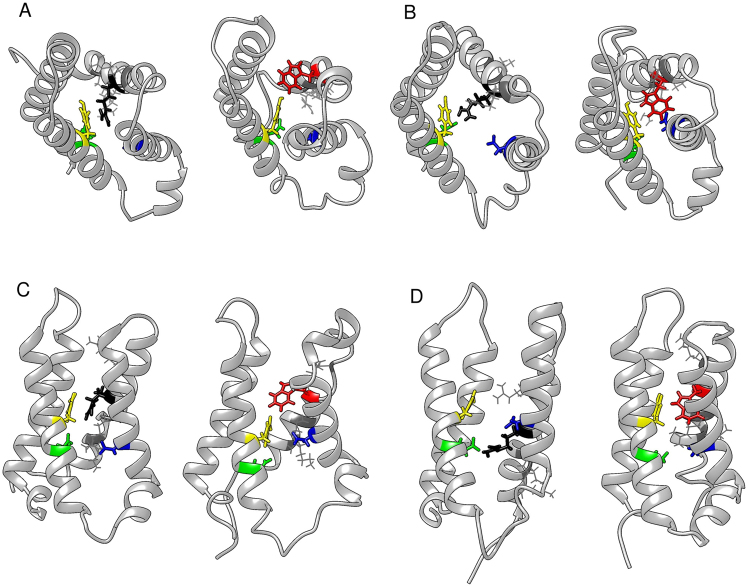
W222 distrupts interactions within the gating charge transfer center. As R222 (black) or W222 (red) enters the gating pore (**A** top view, **C** side view), the native arginine appears to interact with Y168 (yellow) while the subsituted tryptophan side chain moves away from Y168. Interaction and distance measurements are recorded in Supplementary Table S3. As R222 or W222 moves down through the gating pore (**B** top view, **D** side view), R222 interacts with E171 (green) across the gating pore, whereas W222 does not cross the gating pore to interact with E171. The gating charge transfer center asparate (D197, blue) does not interact with R222 or W222 in simulation.

After the R222W and wild type models were equilibrated with the residue of interest above the gating pore, a hyperpolarizing force was applied to the membrane. The native arginine moved towards Y168 to occlude the gating pore whereas the substituted tryptophan at R2 rotated towards the hydrophobic lipid environment as it moved into the gating pore (Fig. 7A,C). When the residue of interest was equilibrated within the gating pore and a hyperpolarizing potential was applied, R222 interacted with E171 across the gating pore, while the substituted tryptophan did not (Fig. 7B,D).

We tested whether the large hydrophobic side chain of the substituted tryptophan interacted unfavorably with residues of interest in the gating pore (Y168, E171 and D197). For the wild type model, Van der Waals and electrostatic interaction energies were favorable between R222 and Y168 (Supplementary Table S3). However, in the R222W model, Van der Waals energy between the substituted tryptophan and Y168 were not as favorable and the residues experienced electrostatic repulsion. Electrostatic interaction with E171 was also disrupted by R222W.

## Discussion

Of the two families reported with the R222W mutation, the previously reported family has a severe clinical phenotype that includes respiratory insufficiency during attacks^17^, while the presently reported family has a mild clinical phenotype characterized by episodes of weakness in the extremities. Additionally, both families contain affected and unaffected mutation carriers, suggesting incomplete penetrance of the mutation, an effect described previously for DII-S4:R2 HypoPP mutations R672S^23^ and R672H^24^.

The observed diversity in phenotype for individuals with identical DI-S4 *SCN4A* mutations reported here and previously^17^ suggests that multiple factors likely play a role in the extent of muscle fiber paralysis. If we assume that R222W muscle fibers contain 50% mutated channels, an assumption that is consistent with an autosomal dominant disease^25^, our electrophysiological measurements show that at the observed −75 mV resting potential of patient muscle fibers, 51% of sodium channels are available for activation. Given that the index patient exhibited episodic weakness but did not experience paralysis with fibers at this resting potential, we can conclude that this amount of channel inhibition does not abolish action potential initiation and elicit paralysis. However, depolarization by only −10 mV would bring channel availability to 37%, reducing the probability of action potential firing^26^. Additional mutational or epigenetic variation (possibly affecting Kir2.1 channels or Na/K ATPase activity) may contribute to putative differences in muscle fiber depolarization in affected versus unaffected family members and may be requisite to promote the full paralytic phenotype as reported for one individual with the R222W mutation^17^.

The DI-S4: R222W mutation enhances both fast and slow inactivation, and attenuates action potentials in patient muscle fibers. Other *SCN4A* HypoPP mutations including DII-S4: R672G/H/S^6,23^ or DIII-S4: R1135C/H^15^ also enhance inactivation, and action potentials from patient muscle fibers harboring R672G^6^ or R1135H^15^ are attenuated. While our findings suggest that effects of R222W on fast and slow inactivation are more pronounced than those observed for DII-S4 or DIII-S4 mutations, it should be noted that our results were obtained with the *Xenopus* oocyte expression system, unlike previous studies on HypoPP sodium channel gating. In a recent report in which R222W was expressed in human embryonic kidney 293 cells, tryptophan substitution at DIS4: R222 did not affect the probability of inactivation, and slowed entry into fast inactivation^27^. Results from studies utilizing different expression systems and recording configurations renders comparison of the magnitude of effects of HypoPP mutations across domains intractable at present. Nevertheless, in the present study we compared the effect of R222W on simulated skeletal muscle fiber action potentials to those obtained from patient action potential recordings. The comparison between fibers housing WT or 50% R222W mutant channels in simulation was based exclusively on our empirically-derived gating parameters for fast inactivation. Notably, our simulations accurately reiterated the attenuation of muscle fiber action potentials in the index HypoPP patient and suggest that stabilization of the fast-inactivated state is causal to the effect of R222W on these action potentials.

The effect of R222W on action potentials was more severe in simulation than in muscle fiber recordings. However, our finding that peak sodium current amplitude was reduced in mutant channels suggests that functional expression of R222W might be impaired *in vivo*, reducing the impact of a loss of function mutation. Our simulations assuming 50% mutated channels would therefore predict a more severe phenotype than that seen in patient recordings. Previous studies have shown that reduced mutant sodium channel expression disrupts the ratio of wild type to mutated channels and contributes to the overall disease phenotype^25,28^. Nevertheless, while the relative reduction in peak sodium current amplitude observed for R222W compared to hNa_V_1.4 is similar in studies using mammalian expression^27^ or *Xenopus* oocytes (this study), it is uncertain whether this effect is also overt in patient muscle fibers and thus contributes to membrane hypoexcitability.

R222W does not affect the probability of sodium channel activation. The finding of a recent report showing that the R222W mutation expressed in mammalian cells does not shift the midpoint of G/V relations^27^ is in agreement with our findings comparing the effects of wild type and R222W channels in the IFM/QQQ background, expressed in *Xenopus* oocytes. Specifically, R222W produces a right shift of the G/V curve in the native background as an indirect consequence of enhanced closed-state fast inactivation, since activation probability is equivalent for R222W and wild type channels in the IFM/QQQ background. However, we also found that R222W/QQQ slows both activation and deactivation, suggesting that steric hindrance of the bulky tryptophan side chain as it moves through the gating pore alters S4 movement in response to either depolarization or hyperpolarization. Slowed activation elicited with tryptophan substitution at DIS4:R2 may contribute to the observed slow rate of rise of action potentials in R222W patient muscle fibers.

It has been proposed that a proton- or cation-selective omega current is a general feature of S4 mutations in type I or type II HypoPP^1,2,4,8,9^. Our measurements of leak current show that R222W promotes inward sodium current with an amplitude and over a range of hyperpolarized voltages similar to that recently reported for this mutation^27^. Hyperpolarization-elicited gating pore currents are also observed in analogous Na_V_1.4 HypoPP S4 mutations at R2 in domains II and III^11,13,20^. This predominant inward omega current overpowers the small outward potassium current produced by inwardly rectifying potassium channels and causes a depolarizing shift in membrane potential from P_1_ to P_2_^7,29^. Recordings from muscle fibers from the index patient of the present study showed a greater density of fibers in the depolarized (P_2_) state compared to controls, and this density shift of fiber potential towards P_2_ was markedly increased with lowered extracellular potassium. From our electrophysiological recordings, it would be expected that inward omega (sodium) current promoted by R222W is observed at the normal resting potential and is more pronounced with hyperpolarization in the setting of hypokalemia.

Although the emergence of omega current upon substitution of an S4 arginine with tryptophan, an amino acid with a bulky side chain, is surprising, we note that R to W mutations associated with type II HypoPP (DII-S4:R3 in hNa_V_1.4^14^) or with arrhythmic dilated cardiomyopathy (DI-S4:R3 in hNa_V_1.5^30^) also promote cationic omega current that contributes to the pathogenesis of disease. Our molecular dynamics simulations showing that hNa_V_1.4-R222W S4 translocation during hyperpolarization results in an unfavorable interaction of the aromatic residue within the gating charge transfer center are similar to molecular dynamics results for hNa_V_1.5-R225W^30^.

In addition, our simulations reveal a strong electrostatic interaction of S4-R222 with S2-E171 during hyperpolarization that may play a role in occlusion of the gating pore. The R222-E171 interaction is predicted in simulation for Na_V_1.4^21^ and disruption of the analogous interaction with substitution of a bulky side chain at R2 (R1239H in Ca_V_1.1 in type I HypoPP^22^) generates a gating pore permeation pathway in simulation. In our molecular dynamics simulations, electrostatic interactions in the gating charge transfer center involving Na_V_1.4-R222 are absent with tryptophan substitution, which may provide the permeation pathway for sodium during hyperpolarization. Thus, our findings from recordings of leak current and molecular dynamics simulations are consistent with the interpretation that R222W promotes an omega current responsible for the depolarizing effect observed in skeletal muscle fibers of the index patient.

Sodium channel fast inactivation is dictated by activation of the DIV voltage sensor^31^. Not surprisingly, hNa_V_1.4 channelopathy mutations in this domain have significant effects on inactivation. Paramyotonia congenita mutations at DIV-S4:R1 (R1448C/H/P/L/S^32^) destabilize the fast-inactivated state exhibited as slowed entry and accelerated recovery, while congenital myasthenic syndrome mutations at DIV-S4:R3 (R1454W^33^) or DIV-S4:R4 (R1457H^34^) stabilize the fast-inactivated state with a hyperpolarized midpoint and slowed recovery, in addition to promoting slow inactivation. Interestingly, mixed paramyotonia/periodic paralysis phenotypes with mutation at DIV-S4:R2 (R1451L^35^) are accounted for by slowed entry and accelerated recovery from fast inactivation, and a hyperpolarized midpoint of fast and slow inactivation.

For hypokalemic periodic paralysis mutations in the S4 segments of domains II or III, general effects include stabilization of fast and slow inactivation, and slowed recovery, also observed in the present study with DI-S4:R222W. Thus, while the role of DIV-S4 in promoting inactivation is well established, S4 movement in other domains also contributes to sodium channel inactivation. Chanda *et al*.^36^ showed that DIV-S4 movement is coupled to S4 translocation in domains DI to DIII, with the highest cooperativity occurring between DI-S4 and DIV-S4. While the mechanism of S4 cooperativity remains uncertain, our finding that R222W significantly enhances fast inactivation raises the possibility that R222 is a determinant in coupling of DI-S4/DIV-S4 translocations that dictate sodium channel inactivation.

## Materials and Methods

### Patients and *in-vitro* studies on native muscle fibers

Whole EDTA (ethylene diamine tetraacetic acid) blood was taken from patients, with informed consent, for *SCN4A* mutation screening. Complete gene sequencing was performed using PCR amplification and Sanger sequencing as described previously^6,37^. Patient studies were approved by the institutional review board in Ulm (Ethics Commission of Ulm University) and conducted according to the Declaration of Helsinki.

Muscle specimens were removed with informed consent from the quadriceps muscles of the index patient of family HypoPP5 (R222W) and three adult individuals with no neuromuscular disease under regional anesthesia. Muscle specimens were about 28 mm in length and 5 mm in diameter. They were further prepared into small bundles and allowed to stabilize over 2 h in a solution also used for resting membrane and action potential measurements. This solution contained 108 mM NaCl, 4 mM KCl, 1.5 mM CaCl_2_, 0.7 mM MgSO_4_, 26.2 mM NaHCO_3_, 1.7 mM NaH_2_PO_4_, 9.6 mM Na-gluconate, 5.5 mM glucose, 7.6 mM sucrose, 290 mosmol/L, maintained at 37 °C, with pH adjusted to 7.4 by bubbling with 95% O_2_ and 5% CO_2_. In some experiments, extracellular K^+^ was decreased to 1 mM.

Membrane potentials were measured using a voltage amplifier (Turbo TEC-05, NPI Electronic Instruments, Tamm, Germany) and glass microelectrodes filled with 3 M KCl and input resistances of 5 to 10 MΩ. Histograms of the potentials were smoothed by density estimation (weighted averaging of rounded points). Parameters were obtained from a Gaussian fit for each peak according to f(x) = y0 + (A/w*sqrt(π/2))*exp(−2*((x-xn)/w)^2) where y0 is the minimum asymptote, A is density, xn is midpoint voltage, and w is a width (mV) coefficient value for the peak in question. Membrane potentials exhibited a two or three-peak density distribution of polarized and depolarized fibers. Action potential recordings were performed with a second microelectrode. This electrode delivered a constant current to hold various resting potentials for at least 1 min to promote recovery of voltage-gated sodium channels. Then, action potentials were elicited by short depolarizing pulses.

### Oocyte voltage clamp experiments

Attempts to express the R222W mutation in mammalian cells were not successful. Therefore, we used *Xenopus* oocytes to characterize gating parameters and investigate putative omega currents in the HypoPP mutant. The gene for the alpha subunit of hNa_V_1.4 (*SCN4A*) was housed in pSP64T, and the beta 1 subunit gene (*SCN1B*) in pGH19. The mutation coding for R222W was constructed by incorporating the following mutant oligonucleotide primers:

Forward: CATCTCAGCCCTGAGGACCTTCTGGGTGCTGC

Reverse: GCAGCACCCAGAAGGTCCTCAGGGCTGAGATG

into *SCN4A* with PCR amplification (Quik Change II XL, Agilent Technologies, Santa Clara, CA). We also utilized the *SCN4A* construct coding for the DIII-DIV linker mutation IFM1303QQQ that eliminates sodium channel fast inactivation^18^. Plasmid constructs were linearized with *EcoRI* (alpha subunit) or *HindIII* (beta subunit). SP6 or T3 RNA polymerases (Ambion mMESSAGE mMACHINE, Life Technologies, Carlsbad, CA) were used for *in vitro* transcription of alpha or beta subunit mRNA respectively. These were co-injected into *Xenopus* oocytes at a mass ratio of 1:3 alpha/beta subunit mRNA. Oocytes were cultured in a medium containing 96 mM NaCl, 2 mM KCl, 1.8 mM CaCl_2_, 1 mM MgCl_2_, 5 mM HEPES (4-(2-hydroxyethyl)-1-piperazine ethane sulfonic acid), 1 mM Na pyruvate, with 3% horse serum and 100 mg/L gentamicin sulfate, at 17.5 °C with gentle agitation for 3 to 7 days prior to recordings. All procedures for surgeries and care of *Xenopus* frogs were conducted according to protocols approved by the Institutional Animal Care and Use Committee at Idaho State University, following ALAC guidelines.

Central alpha pore currents were recorded with the cut-open oocyte configuration using a CA-1B amplifier (Dagan Corporation, Minneapolis, MN) and acquired with PatchMaster 2.37 software (HEKA, Bellmore, NY). External bath solution contained 2 mM CaOH_2_, 10 mM HEPES, 115 mM NaOH. The internal solution contained 115 mM N-methyl D-glucamine (NMDG), 10 mM HEPES, 2 mM EGTA (ethylene glycol bis(2-amino-ether) tetra-acetic acid). Oocytes were permeabilized by adding 0.5% saponin in internal solution to the bottom chamber. Glass microelectrodes with resistances of 0.6 to 0.9 MΩ were used for recording membrane potential against ground for voltage clamp. Leak currents were recorded in the same cut-open oocyte configuration. For these experiments, the external solution contained 2.5 mM BaOH to reduce background leak^13^ with Na^+^ or NMDG as the relevant cation. Channels were blocked with 2 µM TTX (tetrodotoxin; Alamone Laboratories, Jerusalem, Israel) after equilibration of oocyte interior for 40 min. All experiments were performed at 20 °C using a HCC-100A amplifier and Peltier device (Dagan). Data were acquired at 10 or 20 µs per point from voltage clamp protocols with leak subtraction using a p/4 protocol (omitted for leak current recordings) and with a holding potential of −120 mV. Sodium currents were analyzed with curve-fitting routines with PulseFit 8.67 and Igor Pro 6.37.

Activation conductance was determined from I/V relations according to equation (1)

1 $${{\rm{G}}}_{{\rm{N}}{\rm{a}}}={{\rm{I}}}_{{\rm{N}}{\rm{a}}}({{\rm{V}}}_{{\rm{M}}}\,{\textstyle \text{-}}\,{{\rm{E}}}_{{\rm{N}}{\rm{a}}})$$

where G_Na_ is sodium conductance, I_Na_ is the peak sodium current amplitude, and (V_M_ – E_Na_) is the driving force (test potential V_M_, empirical sodium reversal potential E_Na_). Normalized current amplitudes from steady-state G/V or inactivation curves were plotted against the conditioning voltage and fitted using a Boltzmann function using equation (2):

2 $${\rm{X}}=1/(1+\exp \{\,-{{\rm{z}}{\rm{e}}}_{0}[{{\rm{V}}}_{{\rm{M}}}\,{\textstyle \text{-}}\,{{\rm{V}}}_{0.5}]/{\rm{k}}{\rm{T}}\})\,{\rm{X}}\in \,\{{\rm{G}}/{{\rm{G}}}_{{\rm{M}}{\rm{A}}{\rm{X}}},{\rm{I}}/{{\rm{I}}}_{{\rm{M}}{\rm{A}}{\rm{X}}}\}$$

where G/G_MAX_ is the normalized activation conductance, I/I_MAX_ is the normalized channel availability, z is the apparent valence, e_0_ is the elementary charge, V_M_ is the test potential, V_0.5_ is the midpoint, k is the Boltzmann constant and T is temperature in K.

Kinetics were calculated by fitting data according to equation (3):

3 $${\rm{I}}({\rm{t}})={\rm{offset}}+{\rm{A}}\,\exp (\,-\,{\rm{t}}/{\rm{\tau }})$$

where I(t) is sodium current as a function of time, A is the asymptote, τ is the time constant for deactivation (t_D_) or inactivation (t_H_), and offset is the plateau asymptote.

### Action Potential Modeling

A two-compartment model of a skeletal muscle fiber was constructed in MATLAB using the Hodgkin-Huxley system of equations^38^ and methods similar to Cannon *et al*.^39^. Briefly, we modeled alteration of sarcolemmal and t-tubule membrane potential as a result of sodium, potassium and nonselective leak currents. Simulated muscle fibers were held at −80 mV until current was applied to stimulate an action potential. Sodium conductance was defined as *m*^3^*h* and potassium conductance was defined as *n*^4^. Voltage-dependent rate constants for sodium channel inactivation (*h*) were calculated from experimentally determined kinetics of fast inactivation entry and recovery. To test the specific effects of inactivation defects, the *h* parameter was the only difference between simulations of fibers containing native or R222W proteins. Gating parameters for sodium channel activation (*m*) and potassium channel activation (*n*) were from Cannon *et al*.^39^ and Filatov *et al*.^40^. However, the k_βm_ parameter (Supplementary Table S1) was adjusted until slope and midpoint of the sodium channel activation G/V curve mimicked those obtained from experiments employing the IFM/QQQ background mutation. Simulated action potentials in a muscle fiber harboring the R222W mutation were created by defining sodium current from equation (4):

4 $${{\rm{I}}}_{{\rm{Na}}({\rm{total}})}=0.5[{{\rm{I}}}_{{\rm{Na}}({\rm{WT}})}]+0.5[{{\rm{I}}}_{{\rm{Na}}({\rm{R}}222{\rm{W}})}]$$

assuming equivalent sodium current contribution from wild type and mutant channels^25,41^. Equations and gating parameters used are summarized in supplementary information.

### Molecular Modeling

An alignment of the top 100 most similar sequences to *SCN4A* was conducted using PSI-BLAST and the Clustal Omega server. Wild type and R222W homology models of hNa_V_1.4 DI were created using MODELLER with the crystal structure of a eukaryotic voltage-gated sodium channel used as the template (PDB 5X0M^42^). The residue of interest (R222 or W222) was positioned above the gating pore (Fig. 7A top view, C side view) or within the gating pore (Fig. 7B top view, D side view) using methods similar to Wood *et al*.^43^. The voltage sensor module (S1–S4) was embedded in a POPC (palmityl-o-phosphatidyl-choline) membrane, solvated with TIP3 (transferable intermolecular potential with 3 points), and ionized with 0.15 M NaCl using VMD (visual molecular dynamics) following methods similar to Groome *et al*.^15^. A CHARMM36 force field was used for the protein and the membrane. NAMD 2.9 (not another molecular dynamics) was used to run molecular dynamics simulations. The system was minimized for 1000 steps and lipid tails allowed to relax for 2 ns, followed by equilibration with the protein restrained for 6 ns. The entire system was allowed to relax until the RMSD (root mean square deviation) of the alpha carbons reached its asymptote. Hyperpolarization was simulated by applying a constant electric field perpendicular to the membrane to move R222 or W222 into, or through the gating pore. Trajectory analyses were completed using PLUMED, VMD RMSD calculator and NAMD Energy plug-ins.

### Statistical analyses

Two-tailed Student’s *t*-tests were used to determine significant differences between data sets with equal variances, and two-tailed Mann-Whitney U tests were used when comparing data sets of unequal variances. An f-test of equality of variances was used to determine which test to use. Data are displayed as mean ± standard error of the mean (SEM) unless otherwise noted, and criterion for statistical significance was set at P < 0.05. For goodness-of-fit tests used to evaluate the fit of models, the chi-squared value was reported.

### Data availability

The datasets generated and/or analyzed during this study are available from the corresponding author upon reasonable request.

## Electronic supplementary material

Supplementary Information
