# Supplementary material for: NaV1.4 DI-S4 periodic paralysis mutation R222W enhances inactivation and promotes leak current to attenuate action potentials and depolarize muscle fibers
Source: Sci Rep. 2018 Jul 10;8:10372. doi: 10.1038/s41598-018-28594-5 (PMC6039468; doi:10.1038/s41598-018-28594-5)
Supplement: Supplementary file 1 — Supplementary Information [file 41598_2018_28594_MOESM1_ESM.pdf]

$Na_V1.4$ DI-S4 periodic paralysis mutation R222W enhances inactivation and promotes leak current to attenuate action potentials and depolarize muscle fibers

Landon Bayless-Edwards[1], Vern Winston[1], Frank Lehmann-Horn[2,4], Paula Arinze[1], James R Groome[1*], Karin Jurkat-Rott[3]

[1]Department of Biological Sciences, Idaho State University, 83209 Pocatello ID, USA
[2]Department of Applied Physiology, Ulm University, 89081 Ulm, Germany
[3]Department of Neuroanesthesiology, Clinic for Neurosurgery, Ulm University, Guenzburg, Germany
[4]Deceased

Supplemental Information

Action potential modeling

A two-compartment model of an action potential in a skeletal muscle fiber was constructed using methods similar to Cannon *et al.*[39] The change in membrane potential over time was defined as in supplementary equation S1:

(S1) $dV/dt = g_{Na}*(V_t - E_{Na}) + g_k*(V_t - E_k) + g_i*(V_t - E_i)$

where g (conductance) is defined as $g_{Na} = \bar{g}_{Na}m^3h$, $g_K = \bar{g}_K n^4$, and g_i is ionic leak conductance, $\bar{g}$ is the maximum ionic conductance (Supplementary Table S2), V_t is the membrane potential in mV, and E is the equilibrium potential for each ion in mV. The *h* parameter was defined experimentally. A barrier model was constructed using inactivation kinetics by plotting the time constants of recovery, closed-state fast inactivation, and open-state fast inactivation, against voltage. Plots were fit with a Gaussian curve according to supplementary equation (S2):

(S2) $\tau\,(V_M) = 1 / (k_f + k_b)$

where τ (V_M) is the time constant of fast inactivation, and k_f and k_b are the rate equations for forward and backward reactions, respectively. Reaction rates were determined according to supplementary equations (S3) and (S4):

(S3) $k_f = A\exp+(\{[z\delta(V_M - V_{0.5})]\} / kT)$

(S4) $k_b = A\exp-(\{[z\{1-\delta\}(V_M - V_{0.5})]\} / kT)$

where A is the half rate at V_0, z is the apparent valency of the reaction, δ is the fractional barrier distance, V_M is membrane potential and $V_{0.5}$ is the midpoint in mV, T is the temperature in K, and k is the Boltzmann constant. These equations were used to define α_h and β_h, rates of entry into and exit from the inactivated state in the Hodgkin-Huxley system of equations[38]. The h parameter of voltage-gated sodium channel inactivation in the Hodgkin-Huxley model was defined by α_h and β_h using supplementary equation (S5):

(S5) $h = \alpha_h / (\alpha_h + \beta_h)$

The sarcolemma and t-tubules comprise the two compartments of the model. Supplementary equation (S6) was used to calculate the ionic current flowing per unit area of t-tubule:

(S6) $I_{ionic} = \eta_{Na}*g_{Na}*(V_t - E_{Na}) + \eta_k*g_k*(V_t - E_k) + \eta_i*g_i*(V_t - E_i)$

where η is the ratio of t-tubule to surface channel densities. Values from Cannon *et al.*[39] were used to define sodium and potassium t-tubule to surface channel densities. We also modeled the accumulation of potassium in the t-tubules with supplementary equation (S7)[39]:

(S7) $d[K]_t/dt = (\eta_k*g_k*(V_t - E_k) + 0.15*\eta_i*g_i*(V_t - E_i))/(F*\zeta) - ([K]_t - [K]_o)/\tau_K$

where $[K]_t$ is the concentration of potassium in the t-tubules, F is Faraday's constant, ζ is the t-tubule surface area to volume ratio (10^{-6}) and τ_K is the time constant of diffusion of K^+ between the t-tubular space and the extracellular space (350 ms). An increase in the concentration of t-

tubule potassium of approximately 0.2 mM was observed for native fibers (similar to Cannon *et al.*[39]), whereas an increase of approximately 0.1 mM was found in fibers containing R222W channels (Supplementary Fig. S1). This difference may be a consequence of action potential attenuation.

Supplementary Table S1: Gating parameters used in action potential model.

**Parameter**	**Control**	**R222W**	**Citation**
$\bar{\alpha}_m$ (ms^{-1})	1	1	[40]
$\bar{\beta}_m$ (ms^{-1})	2	2	[40]
$\bar{V}_m$ (mV)	-30.5	-30.5	This paper
$K_{\alpha m}$ (mV)	10	10	[39]
$K_{\beta m}$ (mV)	13	13	This paper
$\bar{\alpha}_n$ (ms^{-1})	7	7	[39]
$\bar{\beta}_n$ (ms^{-1})	40	40	[39]
$\bar{V}_n$ (mV)	-40	-40	[39]
$K_{\alpha n}$ (mV)	0.01	0.013	[39]
$K_{\beta n}$ (mV)	0.07	0.07	[39]
$\bar{V}_h$ (mV)	-50.9	-80.9	This paper
g_i (mS/cm^2)	0.75	0.75	[39]
$\bar{g}_{Na}$ (mS/cm^2)	150	150	[39]
$\bar{g}_K$ (mS/cm^2)	21.6	21.6	[39]
Resting potential (P_1)	-80.0	-80.0	This paper

Supplementary Table S2: Values used in k_F and k_B calculations.

**Parameter**	**Control**	**R222W**
A	0.019	0.007
z	4.54	4.01
δ	0.50	0.35

$V_{1/2}$	-55.1	-80.6
kT	25.3	25.3
χ^2	24.5	53.1

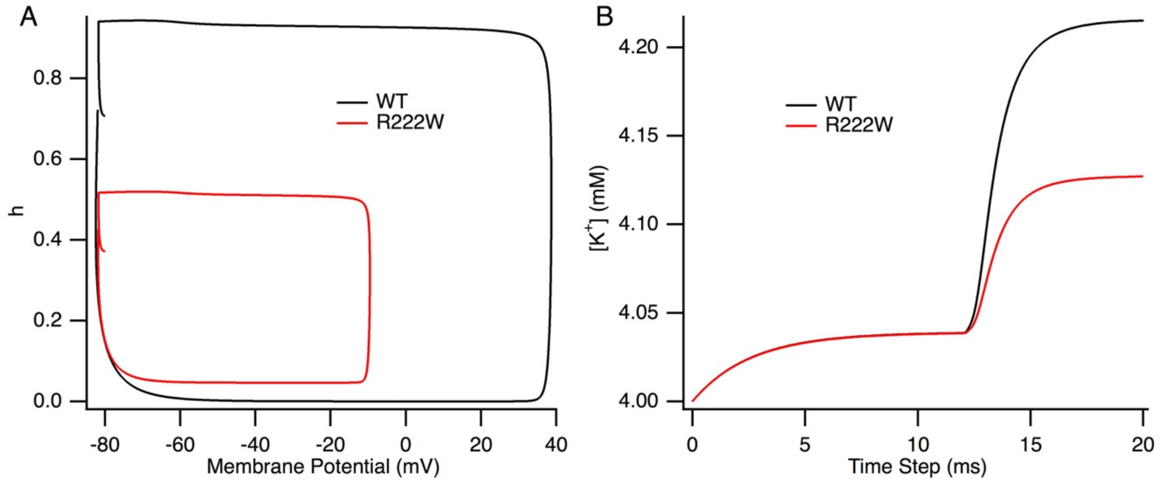

Supplementary Figure S1: Additional parameters modeled in action potential simulations. A phase diagram of the *h*-gate parameter, or the inverse probability of inactivation, is shown in (A). The *h*-gate parameter from the R222W fibers shows a higher inactivation probability, consistent with electrophysiological results. Potassium accumulation in the t-tubules in shown in (B). There is less potassium accumulation after one action potential in R222W fibers compared to wild type.

Supplementary Table S3: Parameters measured in molecular dynamics simulations.

	**VdW (kcal / mol)**		**Electrostatic (kcal/mol)**		**Distance (Å)**	
	R222	W222	R222	W222	R222	W222
Y168	-3.1 ± 0.3	-1,9 ± 0.2	-0.8 ± 1.3	2.1 ± 1.5	4.3 ± 0.2	6.8 ± 0.4
E171	4.1 ± 2.4	-0.1 ± 0.03	-99.6 ± 7.9	-0.5 ± 0.5	6.8 ± 0.6	9.9 ± 0.3
D197	-0.03 ± 0.01	-1.0 ± 0.4	0.1± 0.1	1.2 ± 0.7	9.2 ± 1.2	7.2 ± 0.4
